# Fine-scale population structure of Japanese Helicobacter pylori provides new anthropological and epidemiological insights

**DOI:** 10.1099/mgen.0.001419

**Published:** 2025-06-10

**Authors:** Kohei Tomonari, Batsaikhan Saruuljavkhlan, Ricky Indra Alfaray, Kartika Afrida Fauzia, Osamu Matsunari, Daisuke Chinda, Tadashi Shimoyama, Phawinee Subsomwong, Nagisa Kinjo, Tetsu Kinjo, Yasuo Hanamure, Masato Shinzato, Rumiko Suzuki, Fukunori Kinjo, Shigeru Takigami, Toshio Fujioka, Takeshi Matsuhisa, Junko Akada, Takashi Matsumoto, Yoshio Yamaoka

**Affiliations:** 1 Department of Environmental and Preventive Medicine, Oita University Faculty of Medicine, Yufu, Oita, Japan; 2 Criminal Investigation Laboratory, Oita Prefectural Police, Oita, Oita, Japan; 3 Department of Gastroenterology and Hepatology, School of Medicine, Mongolian National University of Medical Sciences, Ulaanbaatar, Mongolia; 4 Helicobacter pylori and Microbiota Study Group, Institute of Tropical Disease, Universitas Airlangga, Surabaya, Indonesia; 5 Research Center for Preclinical and Clinical Medicine, National Research and Innovation Agency, Cibinong Science Center, Jalan Raya Jakarta-Bogor Km. 46, Cibinong, Bogor 16915, West Java, Indonesia; 6 Oita Nakamura Hospital, Oita, Oita, Japan; 7 Department of Gastroenterology, Hirosaki University Graduate School of Medicine, Hirosaki, Aomori, Japan; 8 Aomori General Health Examination Center, Aomori, Aomori, Japan; 9 Department of Microbiology and Immunology, Hirosaki University Graduate School of Medicine, Hirosaki, Aomori, Japan; 10 Ryusei Hospital, Naha, Okinawa, Japan; 11 Department of Endoscopy, University of the Ryukyus Hospital, Nishihara, Okinawa, Japan; 12 Hanamure Hospital, Ichikikushikino, Kagoshima, Japan; 13 Department of Gastroenterology, Okinawa Prefectural Miyako Hospital, Miyakojima, Okinawa, Japan; 14 Population Genetics Laboratory, National Institute of Genetics, Mishima, Shizuoka, Japan; 15 Center for Gastroenterology, Urasoe General Hospital, Urasoe, Okinawa, Japan; 16 Takada Chuou Hospital, Bungotakada, Oita, Japan; 17 Department of Gastroenterology, St. Marianna University School of Medicine, Kawasaki, Japan; 18 Gastroenterology and Hepatology Section, Department of Medicine, Baylor College of Medicine, Houston, Texas, USA; 19 Research Center for Global and Local Infectious Diseases, Oita University, Yufu, Oita, Japan

**Keywords:** ancestry analysis, gastric cancer, *Helicobacter pylori*, human migration, population structure

## Abstract

*Helicobacter pylori* is considered to contribute to gastric cancer and is also used as a marker to trace human migration due to its co-evolution with humans. To understand the recently proposed tripartite model suggesting three ancestral origins for the Japanese population and address the enigma of the high incidence of gastric cancer in Northeast Hondo (Hondo is mainland Japan), we conducted a fine-scale population structure analysis using a large Japanese *H. pylori* dataset, including 438 strains from 9 regions based on whole-genome sequences. As a result of fineSTRUCTURE analysis, it was found that *H. pylori* in Northeast Hondo is genetically distinct from hspEAsia subgroup 7 (sg7), which is widely distributed elsewhere in Hondo. We named this new subgroup hspEAsia-sg8 (Northeast Hondo). Ancestry analysis using ChromoPainter revealed that, while a large proportion of the genomes of hspEAsia-sg8 strains were painted by donors from their own population, the ancestry components of hspEAsia-sg7 showed a high proportion of Chinese and Korean components, suggesting that they were formed through admixture with continental hspEAsia subgroups. These results align with human genome studies, which indicate an original ancestry component in Northeast Hondo and a higher proportion of East Asian components in West Hondo, supporting the tripartite model. This also suggests novel potential for biogeographic ancestry inference in forensic science, as the *H. pylori* genome can distinguish Hondo populations. Furthermore, fixation index analysis comparing the genome of hspEAsia-sg8 with other Japanese hspEAsia subgroups revealed a high number of nonsynonymous mutations in *hp0378* (*ccsBA*) and *hp0377* (*dsbC*/*ccmG*). Because these genes are involved in cytochrome c maturation and disulphide bond formation, the detected mutations may affect bacterial survival, growth or pathogenicity. This study supports the tripartite model for the formation of modern Japanese people and suggests that the strain of *H. pylori* prevalent in the Northeast Hondo region may contribute to the high incidence of gastric cancer there.

**Impact Statement**

hspEAsia, a subpopulation of *Helicobacter pylori*, is widely distributed in the people of Hondo (mainland Japan). Although hspEAsia distributed in Hondo has been considered genetically homogeneous, the genetic heterogeneity within the Hondo human host population and the geographic gradient in gastric cancer incidence led to the hypothesis of hspEAsia heterogeneity within Hondo. A fine-scale population structure analysis of Japanese hspEAsia based on whole-genome data revealed a genetically distinct subgroup of hspEAsia in Northeast Hondo, a region with elevated gastric cancer incidence. Ancestry analysis revealed that this subgroup possesses its own unique component, in contrast to other regions of Hondo, which was formed through admixture with Chinese and Korean subgroups. This aligns with recent human genome studies proposing the tripartite model, which suggests that modern Japanese people have three ancestral components. Additionally, a higher number of mutations were observed in genes involved in bacterial survival, growth and pathogenicity in hspEAsia subgroups in Northeast Hondo. These findings support the tripartite model and suggest that *H. pylori* distributed in Northeast Hondo may contribute to the high incidence of gastric cancer in this region.

## Data Summary

All supporting data have been provided within the article or through supplementary data files. Newly sequenced data in this study are presented in Table S1 (available in the online Supplementary Material) and deposited as PRJNA1197370. Accession numbers of publicly available genome data are listed in Table S2.

## Introduction

*Helicobacter pylori* is a Gram-negative bacterium that is considered to contribute to various gastric and duodenal diseases such as gastric cancer [1,2]. *H. pylori* has been classified into seven region-specific major populations worldwide, namely, hpAfrica1, hpAfrica2, hpNEAfrica, hpEurope, hpAsia2, hpSahul and hpEastAsia, using multilocus sequence typing based on seven housekeeping genes [3,5]. The pathogenicity of *H. pylori* varies among *H. pylori* populations that possess different genotypes of virulence genes such as *cagA* and *vacA* [6]. Furthermore, this bacterium has been used as a marker to trace human migration [3,7,9], and our recent study suggested that its genome has evolved in response to adaptations to the host’s diet [10]. Therefore, it is important to understand the population structure of *H. pylori* for anthropological, epidemiological and clinical research.

**Fig. 1.**
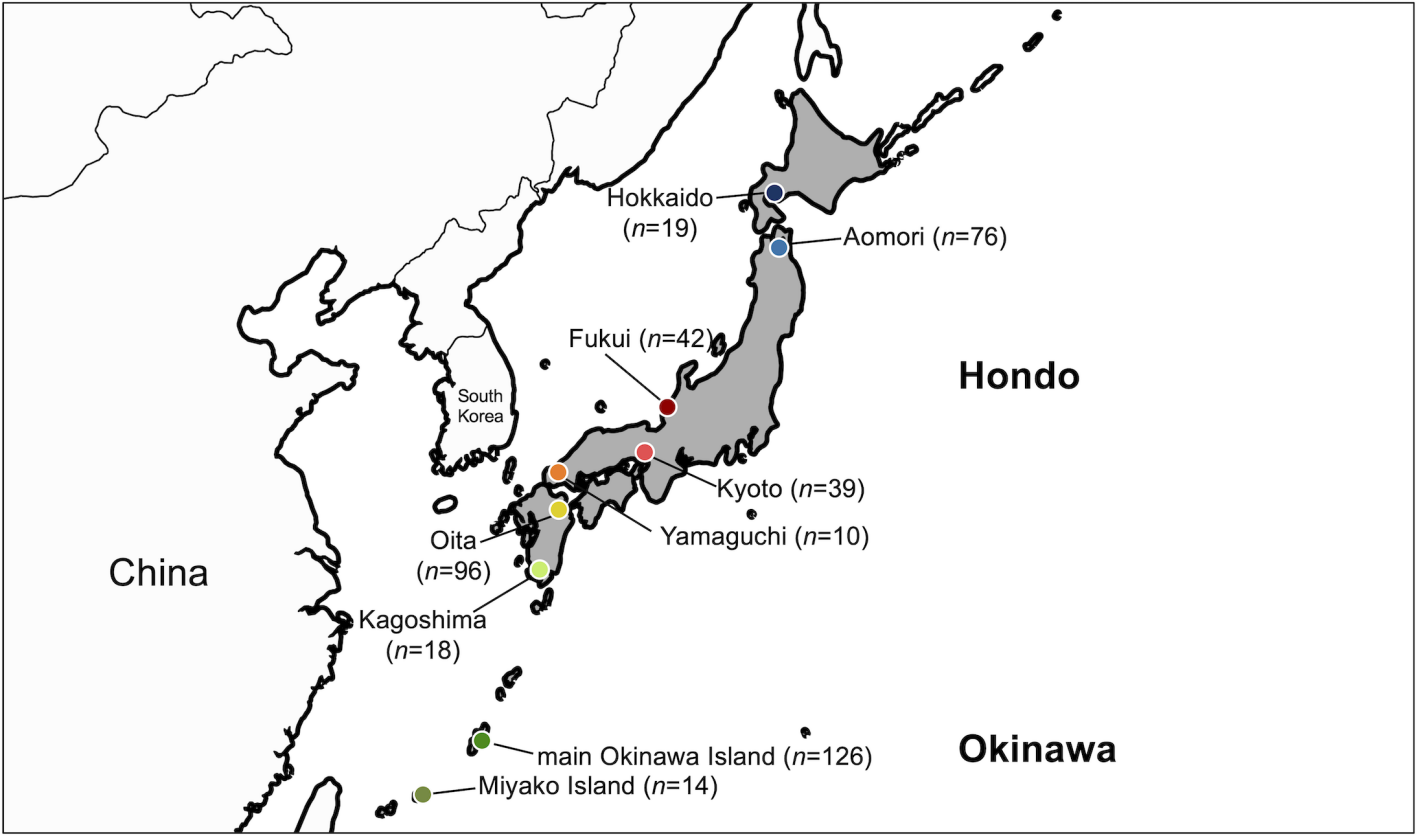
The geographical locations of the Japanese *H. pylori* strains used in this study (*n*=438). Japan is shaded in grey, consisting of Hondo and Okinawa. The numbers in parentheses represent the numbers of strains.

Japan is an archipelago (378,000 km^2^) in East Asia, consisting of Hondo (mainland Japan) and Okinawa (Fig. 1). The most widely supported theory for the origin of the Japanese people is the ‘dual-structure model’ [11]. This model proposes that modern Japanese people were formed through the admixture of indigenous hunter-gathering people (called Jomon people) and rice-farming immigrants from the Asian continent (called Yayoi people) after the start of the Yayoi period (tenth century B.C. to third century A.D.) [12]. The Yayoi people expanded their settlements from Western Hondo throughout the whole of Hondo, facilitating admixture with the Jomon people. By contrast, the northern region (Hokkaido) and southern region (Okinawa) of Japan were less influenced by immigrants and preserved a high proportion of Jomon genetic components. As a result, modern Japanese people could be genetically divided into three populations: Hokkaido Ainu, Hondo and Okinawa people [13,15]. However, recent studies in human genetics suggest that there is also a genetic substructure within the Hondo people [12,16]. Recently, two human genome studies have proposed a new view regarding Japanese origins called the ‘tripartite model’ [17,18]. These studies suggested an additional migration from East Asia after the start of the Kofun period (third to seventh century A.D.). Since the dynamic evolution of the *H. pylori* genome in the Americas within 500 years after European colonization has been revealed [19,20], it may also provide insights into this new genomic evidence.

From clinical perspectives, Japan has the second-highest incidence of gastric cancer in the world [21] and, within Japan, Northeast Hondo is a particularly high-risk region, with notably high incidence and mortality rates for gastric cancer [22]. This region is known for its distinct food culture, including high salt intake, which has been thought to contribute to the high risk in this region [23,24]. However, given recent findings on the influence of the host’s diet on the evolution of the *H. pylori* genome [10], *H. pylori* in this region may have evolved differently from that in other regions, which may also contribute to the high risk for gastric cancer there. In recent years, we reported that the relatively homogeneous hspEAsia, a subpopulation of hpEastAsia that is widely distributed in China, Korea and Japan could be divided into six subgroups (sg), namely, sg1 (Southwest China), sg2 (Southeast China), sg3 (Northeast China), sg5 (Korea), sg6 (Okinawa) and sg7 (Hondo) (sg4 was not a member of hspEAsia but a Mongolian subgroup) [25]. However, no region-specific subgroup distinctive to the region associated with a high risk of gastric cancer has been identified so far, and its characteristics remain unknown.

This study consisted of two strands involving a detailed population structure analysis of Japanese *H. pylori* using a large dataset, including *H. pylori* from Northeast Hondo. First, we investigated the new evidence on the formation of the modern Japanese population using *H. pylori* genomes. Second, we investigated the characteristics of *H. pylori* distributed in Northeast Hondo, a region associated with a high risk for gastric cancer.

## Methods

### *H. pylori* isolates and DNA extraction

We collected *H. pylori* strains from five regions of Japan (Aomori, Oita, Kagoshima, Miyako Island and the main Okinawa Island) and Korea in this study. Twenty-seven Aomori strains isolated at Hirosaki University Hospital in Aomori were transported to Oita University. Thirty-two main Okinawa Island strains and 44 Korean strains were isolated in previous studies [26,27]. In addition, we collected Japanese strains isolated at Takada Chuou Hospital in Oita (*n*=7), Hanamure Hospital in Kagoshima (*n*=18), Okinawa Prefectural Miyako Hospital on Miyako Island (*n*=14) and University of the Ryukyus Hospital on the main Okinawa Island (*n*=3) between 2022 and 2024. These *H. pylori* strains were cultured from biopsies by SRL, Inc. (Tokyo, Japan), a clinical laboratory company, during the clinical processes of each hospital. These strains were transported to Oita University; then, * H. pylori* strains were subcultured on a Brucella agar plate supplemented with 7% horse blood under microaerophilic conditions. *H. pylori* DNA was extracted using DNeasy Blood and Tissue Kit (QIAGEN, Hilden, Germany). Written informed consent was obtained from each participant, and this study was approved by the Ethics Committee of Oita University Faculty of Medicine (approval number: 2281).

### Whole-genome sequencing and genome assembly

Whole-genome sequencing was performed on 101 Japanese strains and 44 Korean strains in this study. *H. pylori* genomes were sequenced on the Illumina MiSeq platform using the 2×300 bp paired-end protocol. DNA library preparation was performed with the TruSeq Nano DNA High Throughput Library Prep Kit (Illumina, San Diego, CA, USA), in accordance with the manufacturer’s instructions. The quality of the raw reads was checked using FastQC v0.11.9 [28], and low-quality reads and adapters were trimmed using Trimmomatic v0.39 [29] with the SLIDINGWINDOW:4:20 option. *De novo* assembly was performed using SPAdes v3.15.4 [30] with the --careful option, and contigs shorter than 200 bp were removed. The quality of assembled genomes was checked using QUAST v5.2.0 [31]. Seven strains were removed due to a high number of contigs (>400), and one strain was removed because of its large genome size (1.79 Mbp). In total, 137 strains were retained for subsequent analysis (Table S1).

### Data preparation

In addition to 137 newly sequenced strains, we used the publicly available data of 615 genomes, including 344 Japanese strains with defined isolated regions and 271 worldwide reference strains from each representative population characterized in a previous study (Table S2). In total, 752 strains including 438 Japanese strains (Fig. 1) were used in this study. All genomes were annotated using Prokka v1.14.6 with default settings [32]. To extract SNPs in the core genome of all genomes, Snippy v4.6.0 was used with the default settings in this study [33]. In brief, whole-genome alignment was performed using snippy-multi with F57 strain as a reference; then, 275,927 core SNPs were extracted by the snippy-core function. The F57 strain was isolated from a gastric cancer patient in Fukui, Japan, and the complete genome sequence has been determined using whole-genome shotgun sequencing [34]. Previous studies have also used this strain as a representative Japanese strain [25,35]. Therefore, given the aim of this study to investigate the detailed population structure of Japanese strains and analyse those isolated from a high-risk gastric cancer region, we used F57 as a reference strain. Among the output files generated by Snippy, the core SNP data (core.vcf) were used for fineSTRUCTURE analysis, while the core SNP alignment file (core.aln) was used for phylogenetic tree construction.

### Population structure analysis

To explore the population structure of Japanese *H. pylori* in detail, genome-wide haplotype-based fineSTRUCTURE analysis was performed as previously described [36] using fineSTRUCTURE v4 software [37]. The core SNP data generated by Snippy were used to prepare the haplotype file, following the instructions provided on the website [38]. The per-site recombination rate, as specified in a recombination map file, was set to the same recombination rate as in a previous study [36]. The fineSTRUCTURE analysis was performed with ‘-s3iters 200000’ and ‘-ploidy 1’ options. In the analysis, the number of chunks exported from a donor to a recipient was calculated, and the co-ancestry matrix was created in the chromosome painting step. Then, fineSTRUCTURE analysis was performed with 100,000 iterations of both the burn-in and the Markov chain Monte Carlo method to cluster each strain based on a co-ancestry matrix. The results of fineSTRUCTURE analysis were visualized using R code provided on the website [38] and heatmap.2x package in R. The results are presented as a heatmap of the co-ancestry matrix and a dendrogram generated from clustering based on this matrix. Strains that belong to the same group exhibit a high level of haplotype sharing and are clustered together in the dendrogram. Population assignments for each strain were determined based on reference strains assigned to each cluster, as well as the geographic origin of the strains. These reference strains were previously assigned to populations in earlier studies (Table S2). The phylogenetic tree was constructed using approximate maximum-likelihood method-based FastTree v2.1.11 software [39] with 1,000 resamples, based on a core SNP alignment file generated by Snippy and visualized using iTOL [40]. To understand the population structure among hspEAsia subgroups in more detail, principal component analysis (PCA) was performed using strains defined as hspEAsia subgroups by fineSTRUCTURE analysis. SNPs in the core genome were extracted from hspEAsia strains only, using the same Snippy method described in the ‘Data preparation’ section, with F57 as the reference. Preprocessing of the SNP data, linkage disequilibrium (LD) pruning and PCA were performed following the procedures outlined on the GitHub page provided by a previous study [41,42]. Briefly, prior to PCA, only biallelic SNPs located in the coding sequence regions were extracted using R. LD pruning and PCA were carried out using Plink v2.0. The result of PCA was visualized using ggplot2 v3.3.5 package in R v4.1.2.

### Ancestry analysis

To infer the ancestral components of each *H. pylori* strain, we conducted chromosome painting using ChromoPainter v2 [37]. All strains were painted as recipients using donor strains that were randomly selected (*n*=15) from each population, subpopulation or subgroup (Table S3). The input files were prepared following the instructions [43]. In addition to the input files used for the fineSTRUCTURE analysis, a population list file was created to specify donor and recipient populations. The analysis was conducted using ‘-j’ option to indicate haploid data. After this, we performed the ancestral chromosome painting using only hpAfrica2, hpAfrica1, hpNEAfrica, hpAsia2, hpNorthAsia, hspUral and hspEAsia as donors, as previously described [44]. The results of chromosome painting were visualized using ggplot2 v3.3.5 package in R v4.1.2. To compare the ancestral proportion among the hspEAsia subgroups, boxplots were created using ggplot2 v3.3.5 package in R.

### Classification of the Japanese hspEAsia subgroups

To investigate the genomic differentiation among the Japanese hspEAsia subgroups, we conducted a fixation index (Fst) analysis using strains belonging to the Japanese hspEAsia subgroups (*n*=336). Whole-genome alignment against F57 was performed using snippy-multi function in Snippy, with strains from the Japanese hspEAsia subgroups. Snp-sites v2.5.1 [45] was used to extract SNPs from the whole-genome alignment obtained by Snippy, and positions that were present in 80% or more of the strains were retained for downstream analysis to capture accessory genes. We calculated the Fst value of each SNP using PopGenome v2.7.5 package in R [46]. SNPs with an Fst value of more than 0.5 were considered as differentiated SNPs and analysed further as described below. For each gene sequence, it was determined whether significant SNPs were synonymous or non-synonymous (NS) using CLC Genomics Workbench 22 (QIAGEN). A detailed analysis was performed on *ccsBA* (*hp0378*) and *dsbC*/*ccmG* (*hp0377*), both of which contain a high number of NS mutations. For each gene, protein structure data were obtained from the Protein Data Bank (https://www.rcsb.org/) if available or were predicted using AlphaFold3 [47]. Fst values were visualized with gradient colour on the protein structure using PyMOL software (Molecular Graphics System, v2.5.7, Schrödinger, LLC). For these genes, aa sequences with significant Fst values were visualized using Weblogo [48]. In addition, the prevalence of *cag* pathogenicity island (*cag*PAI) genes, which are strongly associated with gastric cancer, was investigated using strains from the Japanese hspEAsia subgroups. Abricate v1.0.1 [49] was used with a 70% identity threshold and 50% coverage to determine the presence of *cag*PAI genes in each strain. The results were visualized as a heatmap using gplots v3.1.3 package in R.

### Phylogenetic analysis of the differentiated genes

The sequences of each gene were extracted from the ffn file obtained by Prokka using blastn from blast v2.12.0 [50], with a 70% identity. Multiple alignment of the gene sequences was performed using MAFFT v7.490 with the --auto option [51]. The gene alignments were manually checked, and incomplete sequences were removed before the tree construction step using FastTree v2.1.11 software [39] and then visualized using iTOL [40].

## Results

### Population structure of Japanese *H. pylori*

**Fig. 2.**
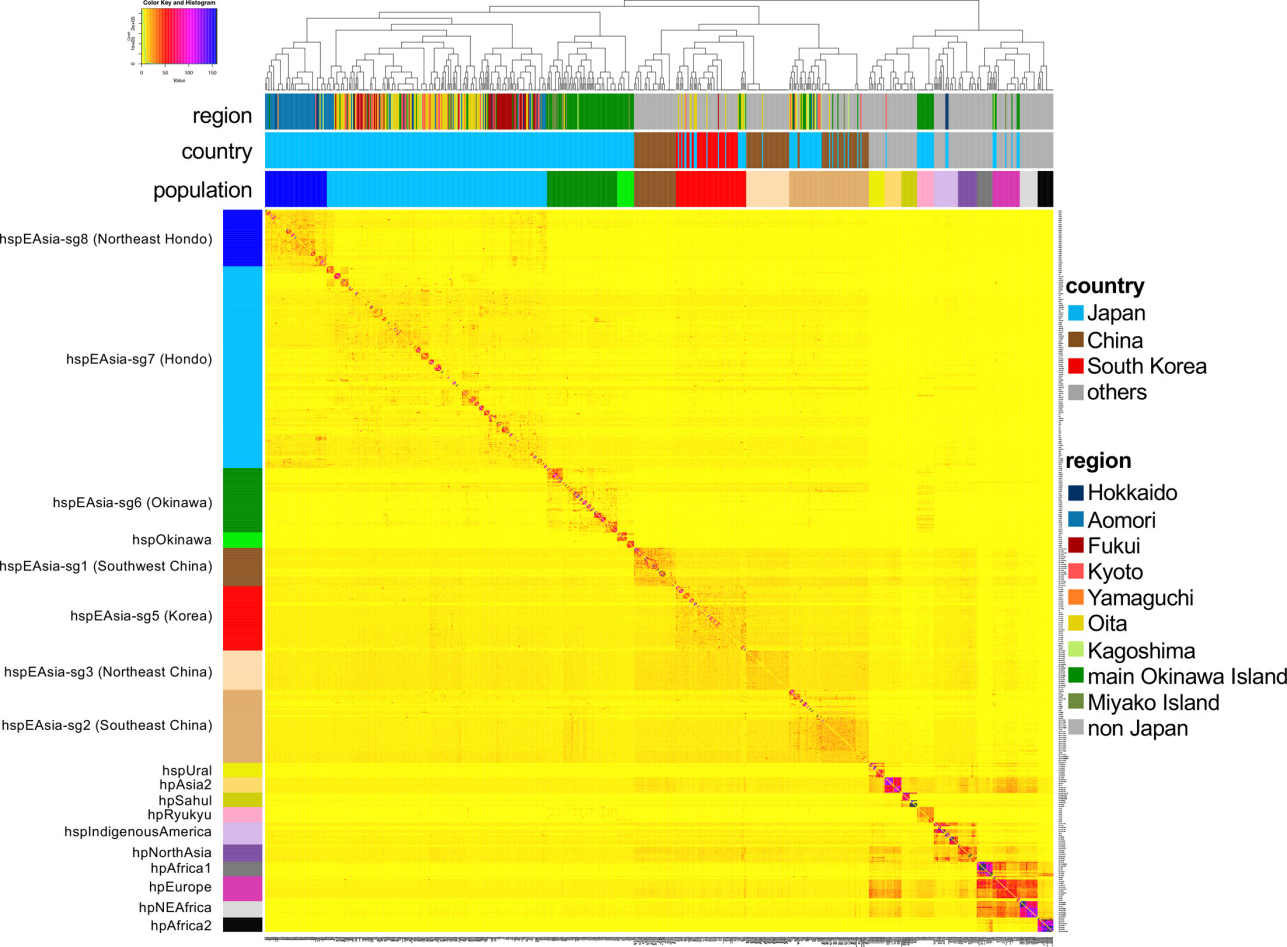
Co-ancestry matrix of all strains used in this study. The heatmap indicates the number of DNA chunks imported from a donor genome (column) to a recipient genome (row). The innermost bar plot represents the *H. pylori* populations, subpopulations or subgroups defined in this study. The isolation country and region of each strain are shown in the middle and top bar plot, respectively.

In this study, we conducted fine-scale population structure analysis using 438 Japanese strains from 9 regions, including previously uncharacterized Northeast Hondo isolates, with a worldwide reference dataset. We identified 18 populations, subpopulations or subgroups based on reference strains (Fig. 2 and Table S3).

The dendrogram was divided into two main clusters: an hpEastAsia cluster and a non-hpEastAsia cluster. The hpEastAsia cluster was further divided into the Japanese cluster and the continental (Chinese and Korean) cluster. Within the Japanese *H. pylori* cluster, in addition to the previously reported hspEAsia-sg6 (Okinawa), hspEAsia-sg7 (Hondo) and hspOkinawa [25,26, 52], a new subgroup has been identified. This newly defined subgroup, consisting predominantly of strains from Aomori, has been named hspEAsia-sg8 (Northeast Hondo). The continental cluster consisted of hspEAsia-sg1 (Southwest China), hspEAsia-sg2 (Southeast China), hspEAsia-sg3 (Northeast China) and hspEAsia-sg5 (Korea), and few Japanese strains were clustered as previously reported [25]. In addition to the hpEastAsia cluster, some Japanese strains were found in the non-hpEastAsia cluster. An Okinawa-specific population, hpRyukyu, was detected within the non-hpEastAsia cluster. Hokkaido Ainu strains were assigned to the hspIndigenousAmerica cluster, and some Japanese strains, mainly from Okinawa, were clustered within hpEurope. The findings of these non-hpEastAsia populations were consistent with previous studies [26,52]. The phylogenetic tree analysis was generally consistent with the result of fineSTRUCTURE analysis (Fig. S1). Although some strains were assigned to different populations in the phylogenetic tree, this discrepancy is likely due to the differing analytical principle of the two methods: fineSTRUCTURE is based on haplotype data, whereas phylogenetic analysis relies on SNP data. In particular, the high recombination rate of *H. pylori* may have contributed to this inconsistency [53].

**Fig. 3.**
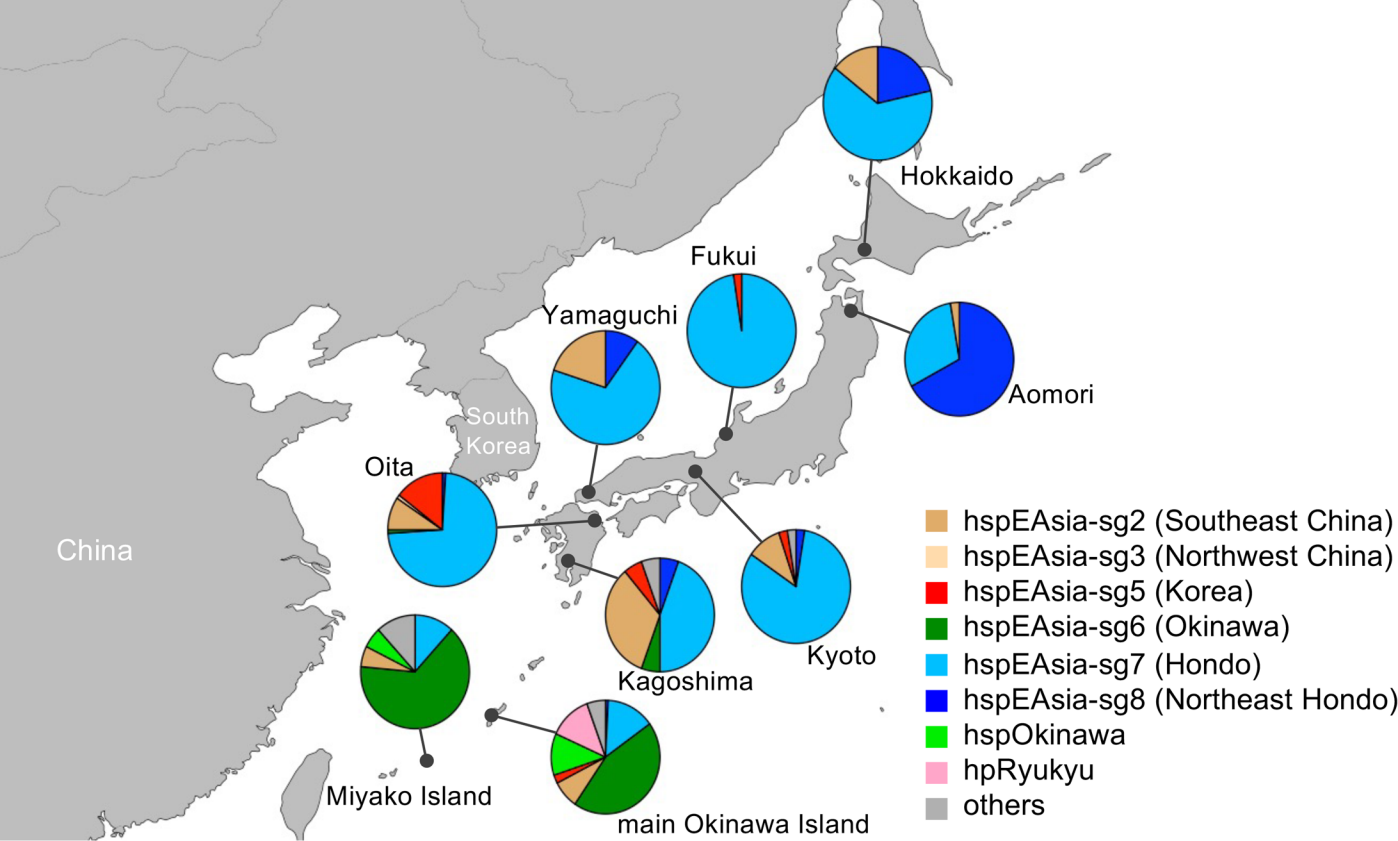
The distribution of *H. pylori* populations within Japan. The colours in the pie chart represent each population.

The identification of hspEAsia-sg8 had a significant impact on the distribution and genomic diversity of the *H. pylori* population across Japan. hspEAsia-sg7 was predominant in Hokkaido, Fukui, Kyoto, Yamaguchi, Oita and Kagoshima. Meanwhile, hspEAsia-sg8 was predominant in Aomori, a region associated with a high risk for gastric cancer, and hspEAsia-sg6 was predominant on the main Okinawa Island and Miyako Island. Furthermore, some strains in Yamaguchi, Oita and Kagoshima, which are located near the Asian mainland, were clustered in the continental hspEAsia-sg2, hspEAsia-sg3 and hspEAsia-sg5 (Fig. 3). Compared with the findings in the Hondo region, numerous populations were observed in the main Okinawa Island, consistent with our previous study [26,52]. Approximately 25% of Okinawa strains were hpRyukyu or hspOkinawa, and 70% were hspEAsia.

**Fig. 4.**
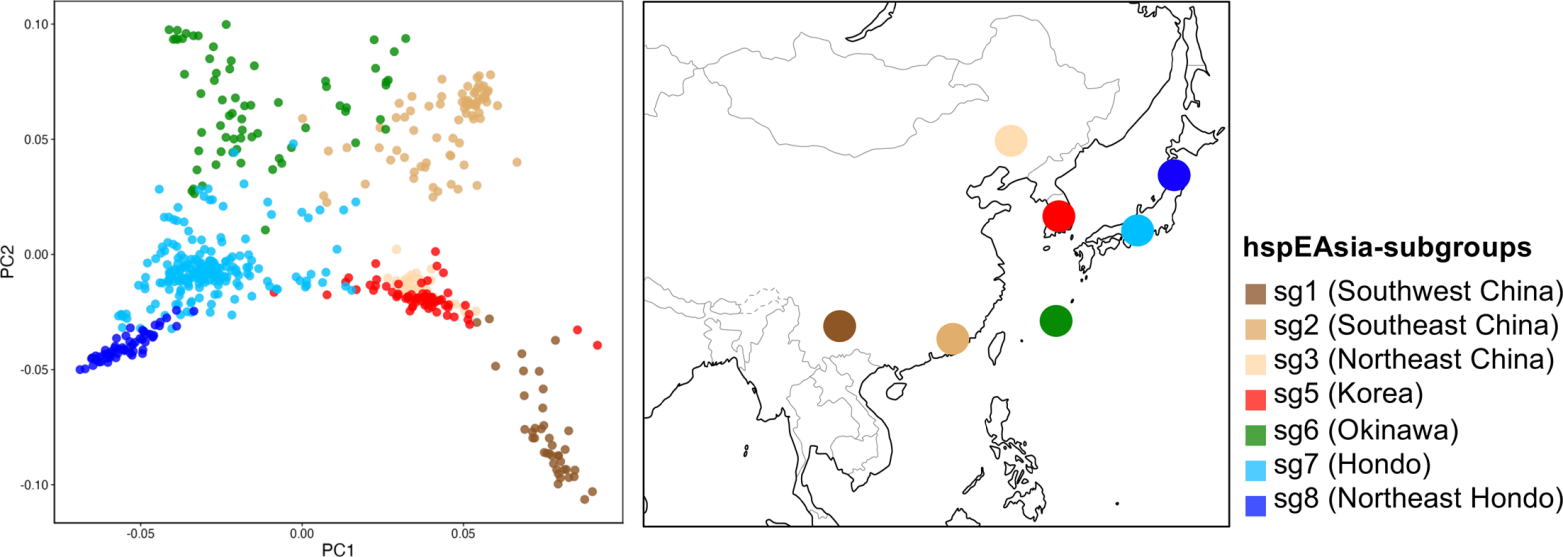
PCA of 560 *H*. *pylori* strains belonging to hspEAsia. Each strain is represented by the colour of the subgroups defined by fineSTRUCTURE analysis. The geographical relationships of each subgroup are represented on the map.

To further explore the relationship among hspEAsia subgroups, we performed PCA using the hspEAsia dataset (Figs 4 and S2). Principal components (PC)1 vs. PC2 reflected the geographical relationships among the subgroups. PC1 correlated with latitude, dividing the Japanese subgroups from the continental ones. Focusing on the Japanese subgroups, each subgroup was divided by PC2, and hspEAsia-sg7 was located between hspEAsia-sg6 and sg8. Among the Japanese subgroups, hspEAsia-sg8 was the furthest from the continental subgroups. Comparing the distribution in each region of Japan, Aomori was the most distant from the continental subgroups, while the western and southern regions tended to be closer to the continental subgroups (Fig. S2).

### Ancestry analysis

**Fig. 5.**
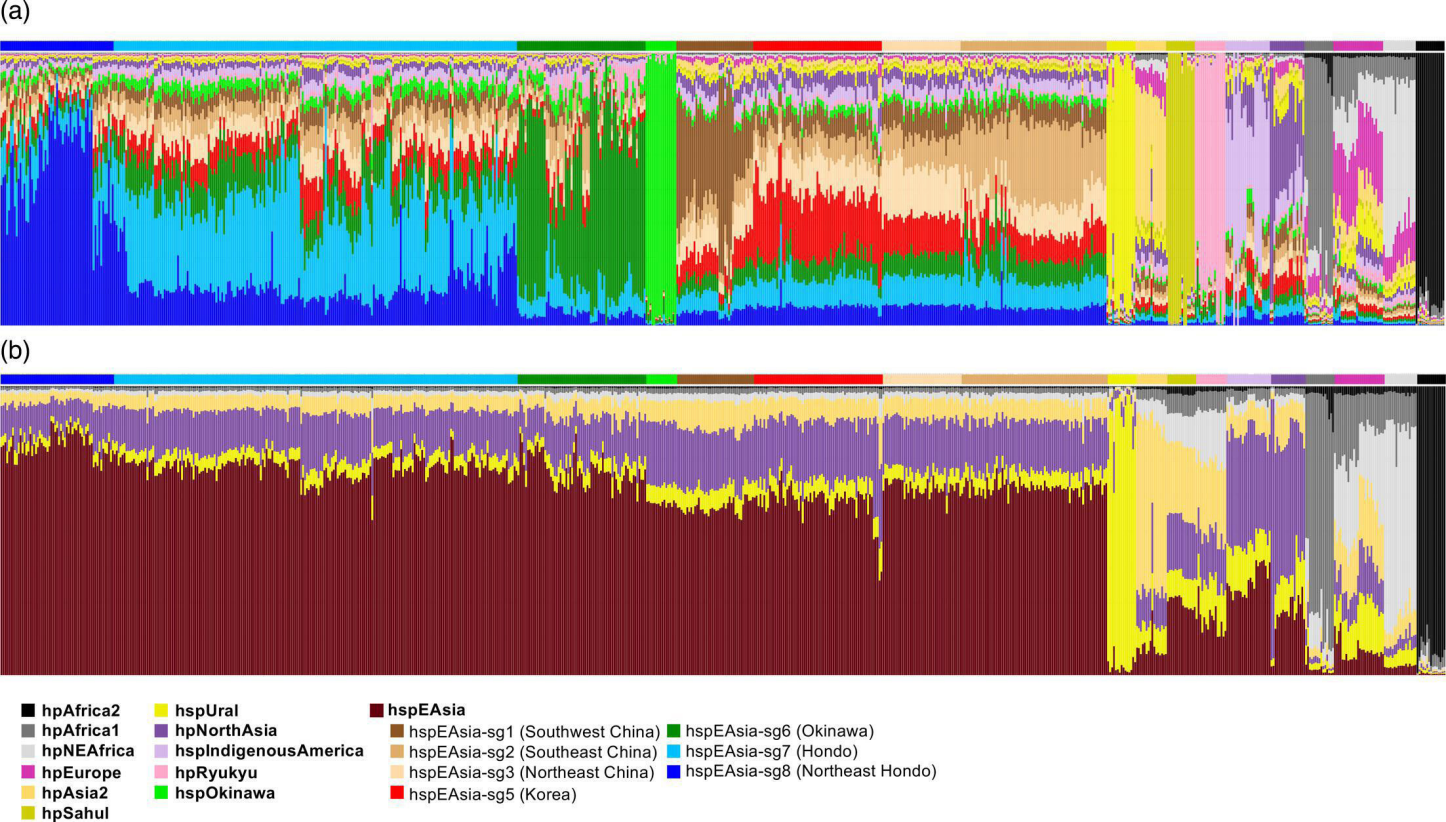
Ancestry of each strain estimated by chromosome painting. Each column shows the proportion of each ancestry, with colors indicating each population. The bar plot above the column indicates the populations defined by fineSTRUCTURE analysis. (a) Full chromosome painting analysis. All populations were used as donors. For hspEAsia, each subgroup was used as donor. (b) Ancestral chromosome painting analysis. Only the true historically ancestral populations, hpAfrica2, hpAfrica1, hpNEAfrica, hpAsia2, hpNorthAsia, hspUral and hspEAsia, were used as donors. The donor strains of hspEAsia were equally selected from all hspEAsia subgroups.

In the Japanese hspEAsia subgroups, a large proportion of the genomes of hspEAsia-sg6 and sg8 strains were painted by donors from their own populations, although a gradient was observed (Figs 5a and S3). Meanwhile, more than 50% of the chromosomes were painted by donors from other populations in most hspEAsia-sg7 strains. In particular, hspEAsia-sg7 had a relatively high proportion of the ancestry components from continental hspEAsia subgroups (sg1, sg2, sg3 and sg5) compared with the other Japanese hspEAsia subgroups (Fig. S4A). As a result of the ancestral chromosome painting using hpAfrica2, hpAfrica1, hpNEAfrica, hpAsia2, hpNorthAsia, hspUral and hspEAsia as donors, the ancestral components of each subgroup within hspEAsia were found to be primarily composed of hspEAsia, hpNorthAsia and hpAsia2, with a slight gradient observed (Fig. 5b). First, the Japanese hspEAsia subgroups had a higher proportion of hspEAsia component than the continental hspEAsia subgroups, with hspEAsia-sg8 exhibiting the highest proportion among them (Figs 5b and S4B). In contrast, the continental hspEAsia subgroups tended to have a higher proportion of hpNorthAsia, hpAsia2 and hpUral components than the Japanese hspEAsia subgroups, and hspOkinawa also showed the same ancestry pattern. Among the Japanese hspEAsia subgroups, the ancestral components exhibited almost the same pattern, although a slight gradient was observed. These findings suggest that the genetic differentiation among the Japanese hspEAsia subgroups is not due to admixture with other populations, but rather to intermixing within hspEAsia subgroups or genetic isolation. In other words, within a small archipelago, while genetically isolated subgroups exist at Northeast Hondo (hspEAsia-sg8) and in Okinawa (hspEAsia-sg6), admixture with continental hspEAsia subgroups has occurred in Central to Western Hondo (hspEAsia-sg7). Interestingly, hspEAsia-sg8 and sg6 correspond to regions with high and low rates of gastric cancer, respectively, suggesting that both components may be involved in the risk of this disease. The genomes of two Okinawa-specific populations, hspOkinawa and hpRyukyu strains, were almost perfectly painted by donors from their own populations, suggesting that these populations have been genetically isolated in Okinawa. The ancestries of hpRyukyu mainly consist of hpAfrica1, hpNEAfrica, hpAsia2, hpNorthAsia, hpUral and hspEAsia. Interestingly, the ancestry components of these two Okinawa-specific populations were also observed in the Japanese hspEAsia subgroups, although the proportion is low.

### Genomic differentiation of *H. pylori* in Northeast Hondo

**Fig. 6.**
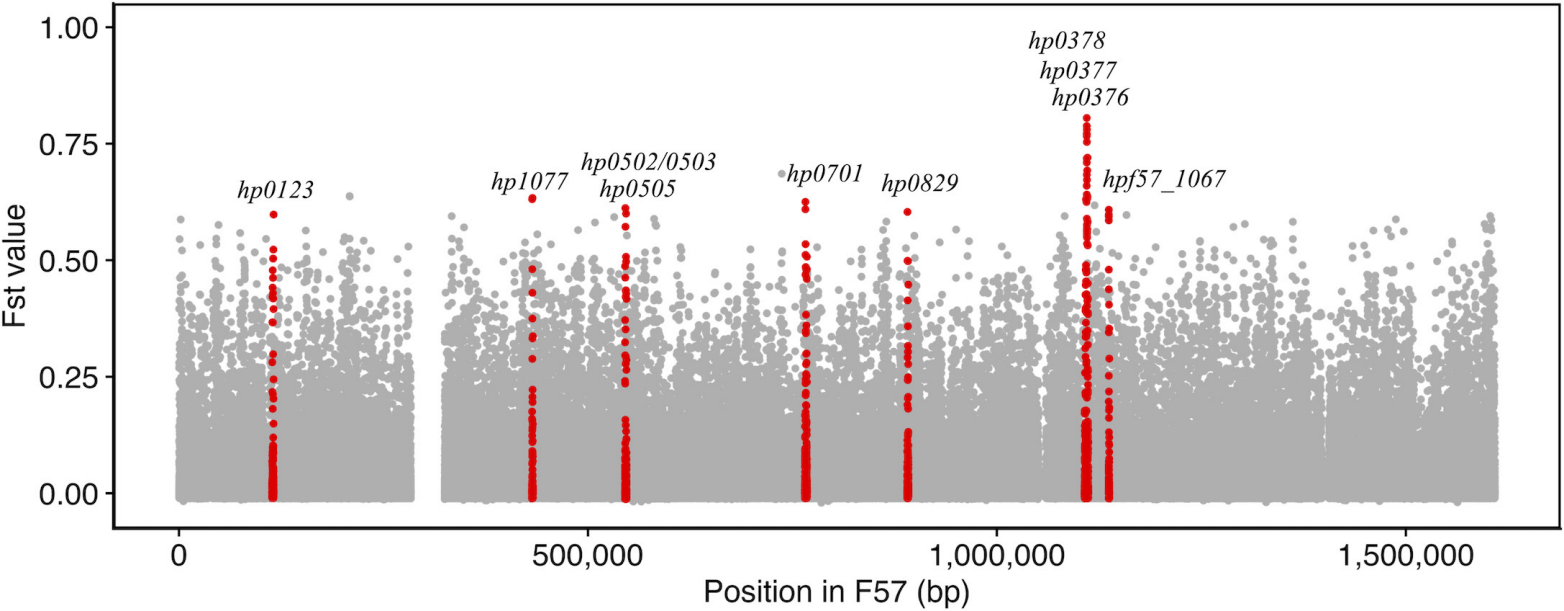
Fst analysis to identify the differentiated genomic regions in hspEAsia-sg8 compared with other Japanese hspEAsia subgroups (sg6 and sg7). The X-axis indicates the nt positions in the F57 reference strain, while the Y-axis shows the Fst values of each SNP. The positions of the top ten genes with the highest Fst values are highlighted in red, along with their gene names.

As a result of fineSTRUCTURE analysis, three hspEAsia subgroups (sg6, sg7 and sg8) were identified within Japan. Of these, hspEAsia-sg6 and sg7 were well characterized in our previous study [25]. Therefore, we focused on the newly identified subgroup hspEAsia-sg8. We performed Fst analysis to investigate genomic differentiation between hspEAsia-sg8 and the other Japanese hspEAsia subgroups (sg6 and sg7). In total, 179 SNPs with Fst value greater than 0.5 were detected in 103 genes. As described below, we focused on the top ten genes with the highest Fst value (Table 1); all detected genes are shown in Table S4 with the details of the mutations. SNPs in the pseudogenes and the intergenic regions were excluded. As shown in Fig. 6, we identified population-specific site variation with high Fst values in the *ccsBA* (*hp0378*), *dsbC/ccmG* (*hp0377*) and *hemH* (*hp0376*) genes. Notably, numerous NS mutations were observed, particularly in *ccsBA* (6 sites) and *dsbC/ccmG* (11 sites) (Table S4). These variations highlight potential functional divergence within these genes between hspEAsia-sg8 and the other Japanese hspEAsia subgroups. Since *hp0377* has been annotated as a multifunctional gene with the functions *dsbC* and *ccmG*, it is referred to as *hp0377* below. The three genes mentioned above are involved in the maturation process of cytochrome c [54]. *hemH* encodes ferrochelatase, which catalyses haem synthesis. HP0377, one of the thiol-oxidoreductases, reduces the oxidized apocytochrome c, and CcsBA plays roles in the transport and ligation of haem to reduced apocytochrome c. In addition, NS mutations were observed in *gyrA*, *M.hpyAXII* and *hpf57_1067. gyrA* is involved in bacterial DNA replication and has been reported to be associated with levofloxacin resistance; however, the NS change position detected in this study was different from previously reported positions related to levofloxacin resistance [55]. *M.hpyAXII* and *hpf57_1067* encode a methyltransferase in the restriction-modification system [56] and glycosyltransferase, respectively.

**Table 1.** The top ten genes with the highest Fst values in the Fst analysis between hspEAsia-sg8 and other Japanese hspEAsia subgroups (sg6 and sg7)

Locus in F57	Locus in 26695	Gene description	Max Fst value	No. of SNPs with Fst >0.5	No. of non-synonymous SNPs (No. of codon change)
HPF57_1039	HP0378	cytochrome c biogenesis protein (*ccsBA*)	0.81	11	7 (6)
HPF57_1040	HP0377	thiol:disulfide interchange protein (*dsbC/ccmG*)	0.78	23	16 (11)
HPF57_1041	HP0376	ferrochelatase (*hemH*)	0.72	3	1 (1)
HPF57_0423	HP1077	high-affinity nickel-transport protein (*nixA*)	0.63	2	0
HPF57_0725	HP0701	DNA gyrase subunit A (*gyrA*)	0.63	5	1 (1)
HPF57_0534	HP0502/HP0503	type Ⅱ restriction-modification system methylase (*M.hpyAXII*)	0.61	3	1 (1)
HPF57_1067	absent	glycosyltransferase	0.61	5	3 (1)
HPF57_0852	HP0829	Inosine 5'-monophosphate dehydrogenase (*guaB*)	0.60	1	0
HPF57_0535	HP0505	R.Pab1 family restriction endonuclease	0.60	2	0
HPF57_0115	HP0123	threonine--tRNA ligase (*thrS*)	0.60	4	0

**Fig. 7.**
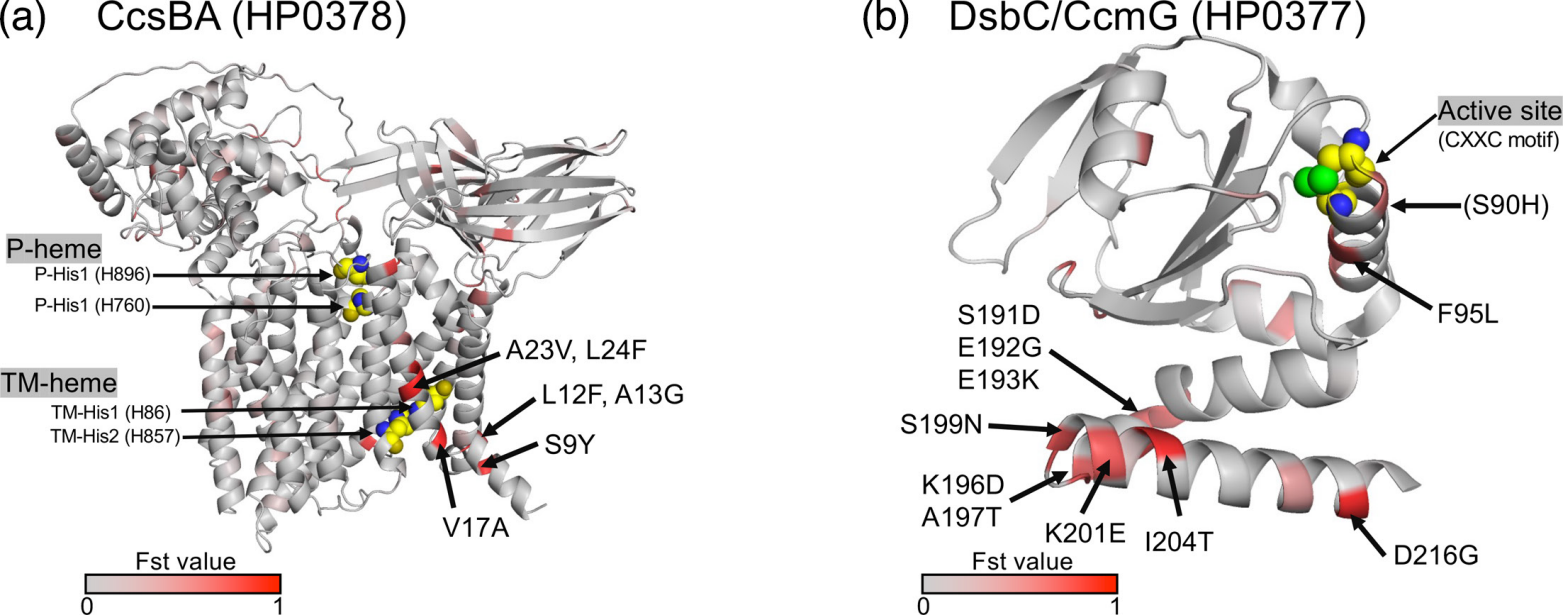
NS mutations fixed in hspEAsia-sg8. Fst values of each position were visualized with gradient colour (grey to red) on the 3D protein structure. The aa number is based on the F57 strain. (**a**) CcsBA (HP0378). The positions of TM-His1, TM-His2, P-His1 and P-His2 are indicated by spheres with carbon (yellow), nitrogen (blue) and oxygen (olive). (**b**) DsbC/CcmG (HP0377). The position of the active site (CXXC motif) is indicated by spheres the same as above and with sulphur (green). I55T mutation was also detected, but the protein structure of this region is not available. The position S90H (Fst 0.42) is indicated in parentheses, as stated in the main text. Compared with HP0377 of 26695, HPF57_1040 of F57 has an insertion of two aa between positions 133 and 134. Therefore, for example, aa 191 in F57 corresponds to aa 189 in strain 26695.

The positions of the NS mutations in CcsBA and HP0377, which include multiple aa changes, were mapped onto the 3D protein structure. Because the protein structure of CcsBA from *H. pylori* was not available, the protein structure was predicted using AlphaFold3, similar to a recent study [57]. In CcsBA, NS changes were located at aa positions 9, 12, 13, 17, 23 and 24 (Figs 7a and S5, Table S4). These mutations were close to the transmembrane (TM)-haem region consisting of TM-His1 (H86) and TM-His2 (H857) where haem initially binds (Fig. 7a). In HP0377, most of the aa changes occurred in the region from aa positions 191 to 204 (Figs 7b and S5, Table S4). Compared with the other Japanese subgroups, aa residues in this region were highly conserved in hspEAsia-sg8. Furthermore, an NS change, F95L (Fst value 0.56), was identified near the active site, CXXC (aa positions 89 to 92). In addition, the second aa of the active site (aa position 90) showed an NS substitution (S90H), although the Fst value was less than 0.5 (Fst value 0.42) (Fig. S5). A global phylogenetic analysis of the *ccsBA* and *hp0377* genes revealed that these genes of the hspEAsia-sg8 strains are phylogenetically closely related to each other (Fig. S6). NS SNPs with Fst >0.5 were detected in major virulence genes, *cagS*, *cagP* and *vacA* (Table S4). In contrast, no SNPs with Fst >0.5 were found in the *cagA* gene. A comparison of the prevalence of *cag*PAI genes among the Japanese hspEAsia subgroups revealed no significant differences (Fig. S7).

## Discussion

To date, whole-genome-based characterization of Japanese *H. pylori* has been limited to only a few regions, such as Fukui, Oita and Okinawa [26,34, 35, 58]. We conducted a fine-scale population structure analysis using a large dataset of 438 Japanese strains from 9 regions in Japan based on whole-genome sequencing data. As a result, we discovered a new hspEAsia subgroup in Northeast Hondo in Japan and named it hspEAsia-sg8 (Northeast Hondo). Downstream analyses provided new insights into the history of human migration to the Japanese archipelago and the enigma of regions associated with a high risk of gastric cancer in Japan.

Unlike European and American populations, which are composed of diverse ethnicities, the Japanese population is formed by a limited number of ancestries. The ancestors of Japanese people are thought to be the indigenous Jomon and the immigrant Yayoi people, and this model has been widely accepted as the ‘dual-structure model.’ However, recent studies have suggested that this model is insufficient to explain the formation of the modern Japanese people. A study based on ancient genomes compared the genomes of Jomon, Yayoi and Kofun (the Kofun period follows the Yayoi period) people, revealing that while Yayoi people were composed of two components (Jomon and Northeast Asian), the Kofun people had an additional East Asian component [17]. The ancestry components of modern Japanese people were found to be generally similar to those of the Kofun period people, suggesting that modern Japanese people were formed through additional admixture with immigrants from East Asia during the Kofun period. They proposed a new model for the formation of the modern Japanese people, the ‘tripartite model.’ Liu *et al*. conducted an ADMIXTURE analysis using whole-genome sequence data of the present-day Japanese population and demonstrated that the modern Japanese could be decomposed into three ancestral components, also supporting the tripartite model [18]. The component suggesting a link to the Jomon was most prominent in Okinawa, while the component indicating a connection to East Asia was most prominent in the West Hondo region. By contrast, the component potentially connected to the ancient population in Japan and the Korean Peninsula was most prominent in Northeast Hondo, where the proportion of the East Asian component was lower than elsewhere in Hondo.

Our data based on the *H. pylori* genomes align to some extent with these studies using the human genome. First, genetically distinct subgroups of *H. pylori* are distributed in Northeast Hondo (hspEAsia-sg8) and other Hondo regions (hspEAsia-sg7). Second, the proportion of the continental hspEAsia ancestry component is lower in hspEAsia-sg8 than in hspEAsia-sg7. HspEAsia-sg8 was mainly distributed in Aomori Prefecture in Northeast Hondo, whereas hspEAsia-sg7 was dominant in other Hondo regions (Fig. 3). HspEAsia-sg7 was also predominant in Hokkaido, the northernmost part of Hondo. This reflects the historical population influx from other Hondo regions to Hokkaido in the modern period. In the ancestry analysis using ChromoPainter, a large proportion of the genomes of hspEAsia-sg8 strains were painted by donors from their own population, whereas the ancestry component of hspEAsia-sg7 contained some degree of continental hspEAsia components (Figs 5 and S3), suggesting that hspEAsia-sg7 was formed through admixture with the continental hspEAsia. In the PCA, hspEAsia-sg7 was close to the continental hspEAsia subgroups, whereas hspEAsia-sg8 was distant from them (Fig. 4). hspEAsia is thought to have spread to Japan during the Yayoi period [52]. Therefore, these findings may reflect the influence of additional migration from East Asia to West and South Hondo after the start of the Kofun period. Liu *et al*. mentioned the Emishi population as a key to the ancestry component of Northeast Hondo [18]. The Emishi historically inhabited Northeast Hondo [59], maintaining political separation from the central government in West to East Hondo until approximately seventh century, when they began to admix with people in these regions [14]. Although the ancestry of Emishi remains a matter of debate, hspEAsia-sg8 may be related to the Emishi. Considering that the ancestry component of Northeast Hondo in the human genome shows potential affinity with the ancient populations of Japan and Korea, and hspEAsia-sg8 has the highest proportion of hspEAsia ancestry component, it is possible that hspEAsia-sg8 represents an early influx of hspEAsia into Japan, which differentiated in Northeast Hondo without admixture with other subgroups. On the basis of these findings, it is considered that the *H. pylori* genome also supports the tripartite model for the formation of the modern Japanese people. However, because we have only used *H. pylori* from one region (Aomori) in Northeast Hondo, further investigation is necessary to elucidate the history of migration in the Japanese archipelago.

As previously reported, diverse populations were distributed in Okinawa. A large proportion of the genomes of hspEAsia-sg6 strains, which we reported in a previous study [25], were painted by donors from their own population. This is presumably a result of genetic differentiation due to geographical isolation and adaptation to Okinawan hosts, which have a different genetic background from the Hondo population. Our ancestry analysis showed that the ancestry components of hspOkinawa strains mainly consisted of hspEAsia and hpNorthAsia. Another specific population, hpRyukyu, was suggested by ancestry analysis to have emerged from the admixture of hpAfrica1, hpNEAfrica, hpAsia2, hpNorthAsia, hspUral and hspEAsia. However, to further understand these populations, a different dataset, including a large dataset of worldwide *H. pylori* strains, is needed. Our previous study suggested that hspOkinawa was formed through admixture with hspMaori (a population that expanded from Taiwan to Oceania) and hspIndigenousAmerica, while the hpRyukyu strains originated in Central Asia [52]. Further analysis involving hspMaori strains and Central Asian strains may provide deeper insights into the population structure of these Okinawa-specific populations. In addition to the Okinawa-specific populations, the Hokkaido Ainu strains were clustered within hspIndigenousAmerica. Our previous study estimated that Japanese populations not classified within hspEAsia diverged in ancient times: hpRyukyu during the Palaeolithic period and hspOkinawa and hspIndigenousAmerica during the Jomon period [52]. For hpRyukyu, it has been hypothesized that *H. pylori* was transmitted without human admixture, possibly carried by Palaeolithic migrants. The two groups with divergence dates in the Jomon period are thought to reflect traces of the Jomon people, persisting in populations such as the Hokkaido Ainu and those in Okinawa, both of which exhibit a high proportion of Jomon ancestry in human genetic studies. Although limited, chromosome painting analyses have also identified ancestry components of these groups within the hspEAsia strains, suggesting traces of admixture with the Jomon people.

The population structure of *H. pylori* genomes in Japan may also have applications in forensic science. In the field of forensic science, inferencing the biogeographic ancestry of unidentified cadavers and traces left by perpetrators is valuable for providing additional information in criminal investigations [60,61]. While previous studies have suggested that Hondo populations could be subdivided using human genome data, such classification typically requires whole-genome data [14,18]. In contrast, the genome of *H. pylori* is relatively small (~1.6 Mb), and genes such as *ccsBA* and *hp0377* may serve as useful genetic markers to distinguish Northeast Hondo from other Hondo regions (Fig. S6). Moreover, several region-specific populations are distributed along the Okinawa archipelago (Fig. 3). This suggests the potential use of the *H. pylori* genome in biogeographic ancestry inference of the Japanese population in forensic science. Because *H. pylori* DNA can be detected in saliva, gastric juice and faeces [62,64], it is feasible to analyse *H. pylori* DNA from forensic samples such as gastric contents, saliva, vomit and faeces. Although the prevalence of *H. pylori* has decreased, particularly among children and young adults in Japan [65], analysis of it would be an option when other useful information is lacking. However, several important limitations must be considered. First, not all individuals are infected with *H. pylori*, making this method inapplicable in such cases. Additionally, because *H. pylori* is typically acquired in early childhood, primarily through family transmission [66], cases in which family members originally carried *H. pylori* from different regions may complicate ancestry inference. Furthermore, emerging evidence of adult-to-adult transmission, as well as zoonotic and zooanthroponotic transmission, should also be considered [67,68]. Therefore, in practice, this method should not be used for precise ancestry identification, but rather as supplementary information in investigations, such as understanding an individual’s or their family’s migration history. Ethical concerns, including the risk of discrimination against specific populations, must also be carefully considered [69].

Northeast Hondo, where hspEAsia-sg8 is predominantly distributed, is known as a region associated with a high risk for gastric cancer. The high incidence of this disease has been attributed to dietary factors such as high salt intake [23,24]. However, this study has revealed that the *H. pylori* distributed in this region is genetically distinct from those elsewhere in Hondo. This result suggests that not only dietary factors but also *H. pylori*-related factors in this region may contribute to the high incidence of gastric cancer. To investigate genomic differentiation among hspEAsia-sg8 and other Japanese hspEAsia subgroups (sg6 and sg7), we conducted Fst analysis on the basis of the core genome. The results revealed SNPs with high Fst values in three genes involved in cytochrome c maturation: *ccsBA* (*hp0378*), *dsbC*/*ccmG* (*hp0377*) and *hemH* (*hp0376*) (Fig. 6). Among these, a significant number of NS SNPs were found in *ccsBA* and *hp0377* (Table 1). Cytochrome c is essential for energy metabolism [70], and its maturation requires the binding of haem to the CXXCH motif of apocytochrome c. Among the pathways for cytochrome c maturation, * H. pylori* uses system II [71]. CcsBA transports haem from the cytoplasm to the periplasm, and cytochrome c is synthesized when the haem binds to the CXXCH motif of apocytochrome c, which has been reduced by HP0377 [72,73]. In this process, haem first binds to the TM haem binding site (TM-haem) of CcsBA and then moves to the external site (P-haem), where it reacts with apocytochrome c [57,73]. In CcsBA, NS changes were observed at aa positions 9, 12, 13, 17, 23 and 24 (Fig. S5 and Table S4). Mapping these mutations onto the 3D protein structure indicates that they are located near the TM-haem site, which may affect the efficiency of haem transport and cytochrome c synthesis, potentially influencing bacterial survival and growth.

HP0377 is one of the disulfide oxidoreductases and is essential for the survival of *H. pylori*; it has also been suggested to function as both CcmG and DsbC [54,74, 75]. The disulphide bond-forming system in *H. pylori* is simple, as it does not encode the classical DsbA/DsbB or DsbC/DsbD but instead relies on HP0231 for the oxidation pathway and HP0377 for the reduction/isomerization reaction [75]. Proper oxidative protein folding is thought to be facilitated by the introduction of disulphide bonds by HP0231, followed by the rearrangement of incorrectly paired cysteine residues by HP0377 [75,76]. The CXXC active-site motif, conserved among thiol-disulphide oxidoreductase protein, is located at aa positions 89–92 in HP0377. NS change was detected at aa position 95 (F95L) near the active site (Figs 7b and S5, Table S4) and also at the second residue in the active site (S90H), although the Fst value was below 0.5 (Fst 0.42). Previous studies have shown that the position corresponding to aa 95 plays an important role in substrate recognition in *Bacillus subtilis* ResA (E80) and StoA (E71) [77,78]. Studies have also reported that the CXXC active site motif is essential for catalytic activity, and mutations in this motif can affect redox properties or play a crucial role in interactions with substrates [79,80]. In *H. pylori*, F95Q was found to hinder the refolding of scRNase [75]. Mutations at these two positions were also detected in a comparison among Colombian subpopulations (P95L, S90H/P) [81] and may be related to the process of adaptation to the human host. Furthermore, more NS changes were observed in aa positions 191–216 in the C-terminal region. This C-fragment is an *H. pylori*-specific atypical extension at the C-terminus compared with CcmG in other bacteria, and a previous study showed that this region is not essential for scRNase isomerization or apocytochrome reduction [75]. However, these mutations alter the polarity and charge of the aa (Fig. S5), potentially affecting protein structure and function. Interestingly, an SNP in *hp0231*, which cooperates with *hp0377*, was detected in our genome-wide association study on gastric cancer based on hspEAsia strains [35]. Many virulence factors of pathogenic micro-organisms contain disulphide bonds, and genes encoding the Dsb system are involved in pathogenicity in East Asian *H. pylori* strains [82,83]. It is possible that genes involved in disulphide-bond formation contribute to the differences in gastric cancer incidence within the East Asian population. In contrast, no significant differences were observed in the prevalence of *cag*PAI genes among the Japanese hspEAsia subgroups (Fig. S7), and no SNPs with Fst >0.5 were detected in *cagA*. However, NS SNPs with Fst >0.5 were detected in several virulence genes (Table S4). In *vacA*, a major virulence factor of *H. pylori*, one NS SNP was identified in the autotransporter domain. Additionally, one NS SNP was found in each of the *cag*PAI component genes *cagS* and *cagP*, although these genes are not essential for either the translocation of CagA or for IL-8 induction [84]. These mutations found in hspEAsia-sg8, which is primarily distributed in regions with a high risk of gastric cancer, may enhance pathogenicity, but further experiments are necessary to elucidate the enigma of regions in Japan associated with a high gastric cancer risk. Furthermore, additional investigation is needed to determine whether this *H. pylori* subgroup has genetically differentiated due to the influence of dietary habits in this region.

## Conclusions

We discovered a new hspEAsia subgroup in Northeast Hondo, Japan, which we named hspEAsia-sg8 (Northeast Hondo). This provides new insights into the formation of the modern Japanese population and supports the tripartite model. Furthermore, genes that differentiated within this subgroup may be involved in pathogenicity, and further investigations could help unravel the enigma of regions in Japan with a high incidence of gastric cancer.

## Supplementary material

10.1099/mgen.0.001419 Uncited Supplementary Material 1.

10.1099/mgen.0.001419 Uncited Supplementary Material 2.

## Abbreviations

Fst: fixation index
LD: linkage disequilibrium
NS: non-synonymous
PC: Principal components
PCA: principal component analysis
sg: subgroup
TM: transmembrane
