# Supplementary material for: Fine-scale population structure of Japanese Helicobacter pylori provides new anthropological and epidemiological insights
Source: Microb Genom. 2025 Jun 10;11(6):001419. doi: 10.1099/mgen.0.001419 (PMC12152251; doi:10.1099/mgen.0.001419)
Supplement: Uncited Supplementary Material 1. [file mgen-11-01419-s001.pdf]

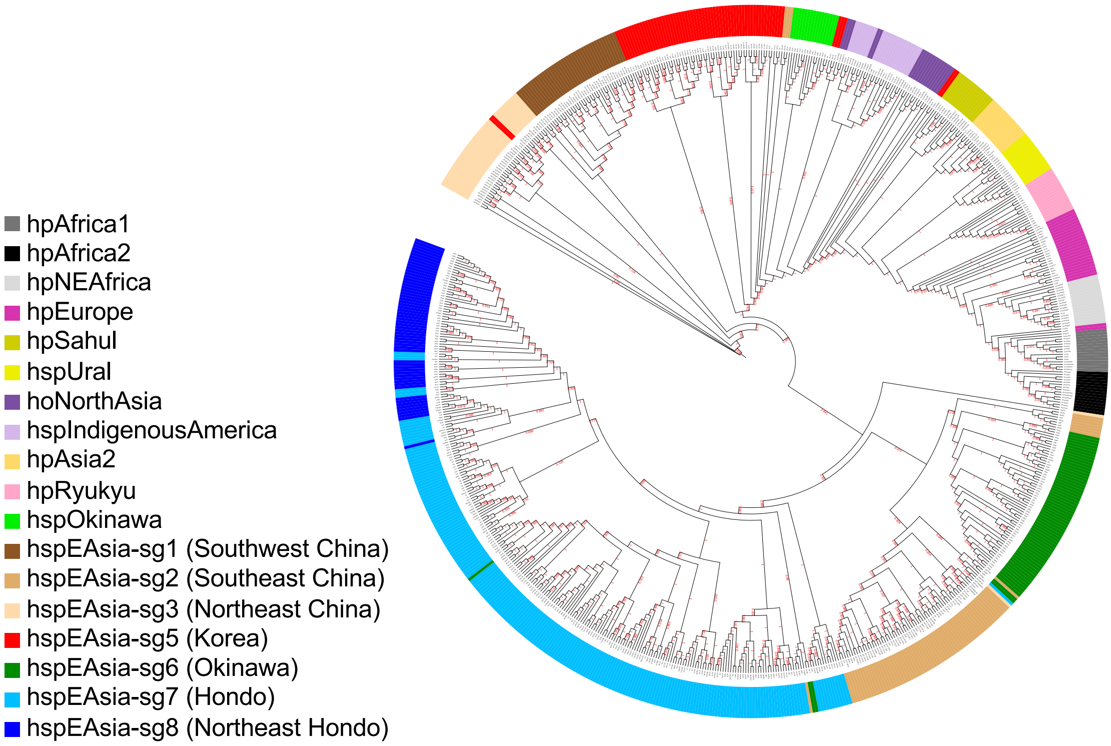

S1 Figure. The phylogenetic tree based on core genome. Numbers at nodes are Shimodaira-Hasegawa test values. Each strip is colored according to the populations defined by fineSTRUCTURE analysis.

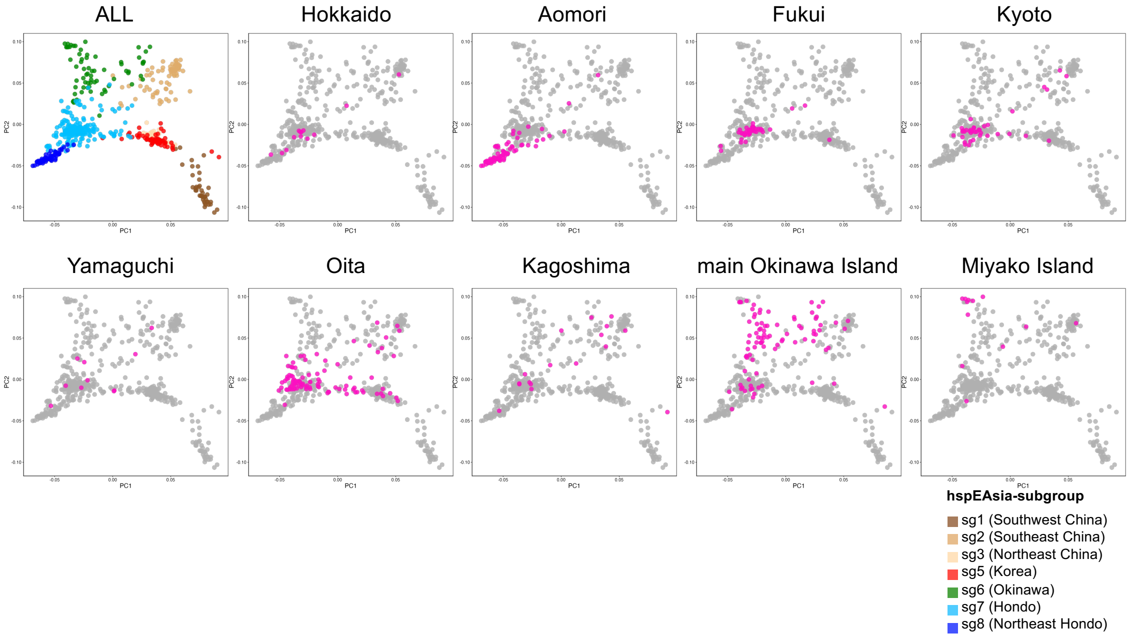

S2 Figure. Regional distribution in the PCA. The pink dots in each plot represent strains isolated from each region.

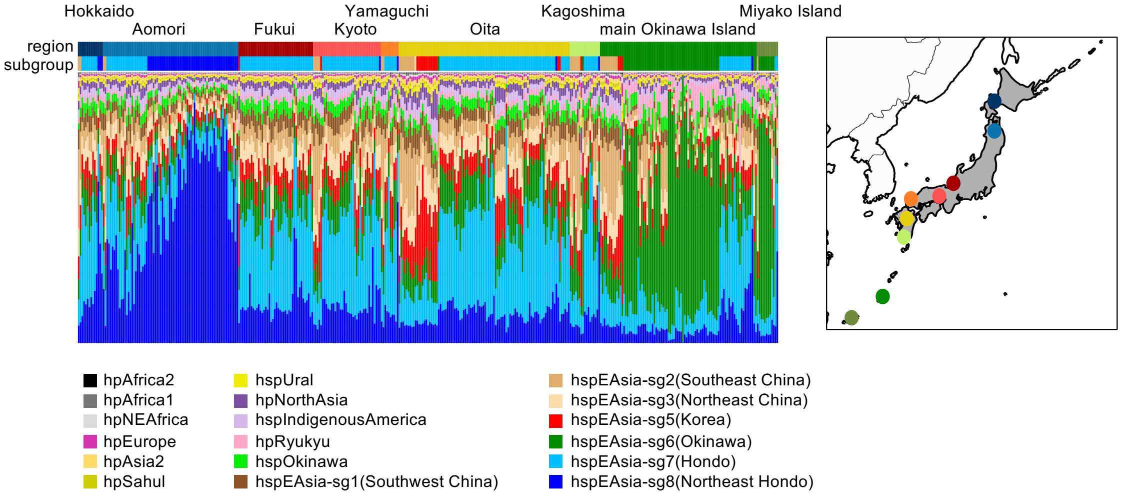

S3 Figure. Ancestral component differences of hspEAsia strains by region in Japan. Each column shows the proportion of each ancestry, with colors indicating each population. The isolated regions of each strain and the subgroups of hspEAsia are shown in the upper and the second bar plots, respectively. The geographical relationships of each subgroup are represented on the map.

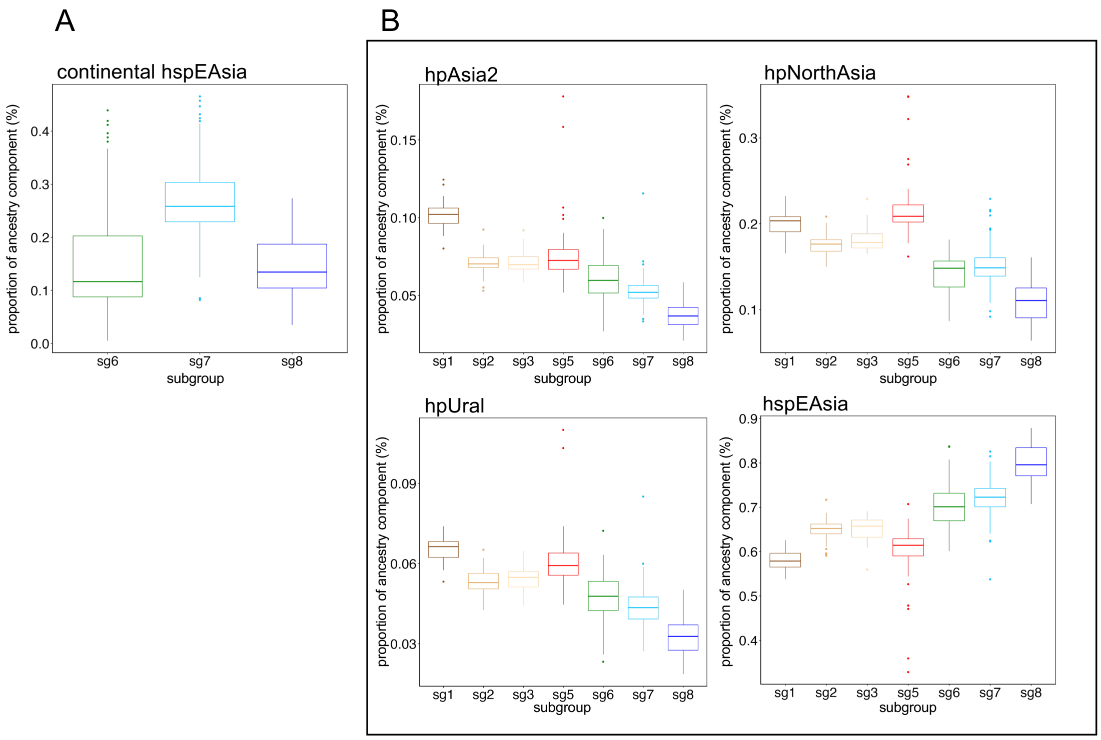

S4 Figure. Comparison of the proportions of each ancestral component in hspEAsia subgroups. In a box plot, the median is represented by a line inside the box. The lower and upper edges of the box represent the 25th (Q1) and 75th (Q3) percentiles, respectively. The whiskers extend to data points within the range of Q1 - 1.5 × IQR (inter quartile range) and Q3 + 1.5 × IQR. Data points outside this range are plotted as outliers. (A) Comparison of continental hspEAsia (sg1-3) components among the Japanese hspEAsia subgroups using full chromosome painting. (B) Comparison of major ancestral components among hspEAsia subgroups using ancestral chromosome painting.

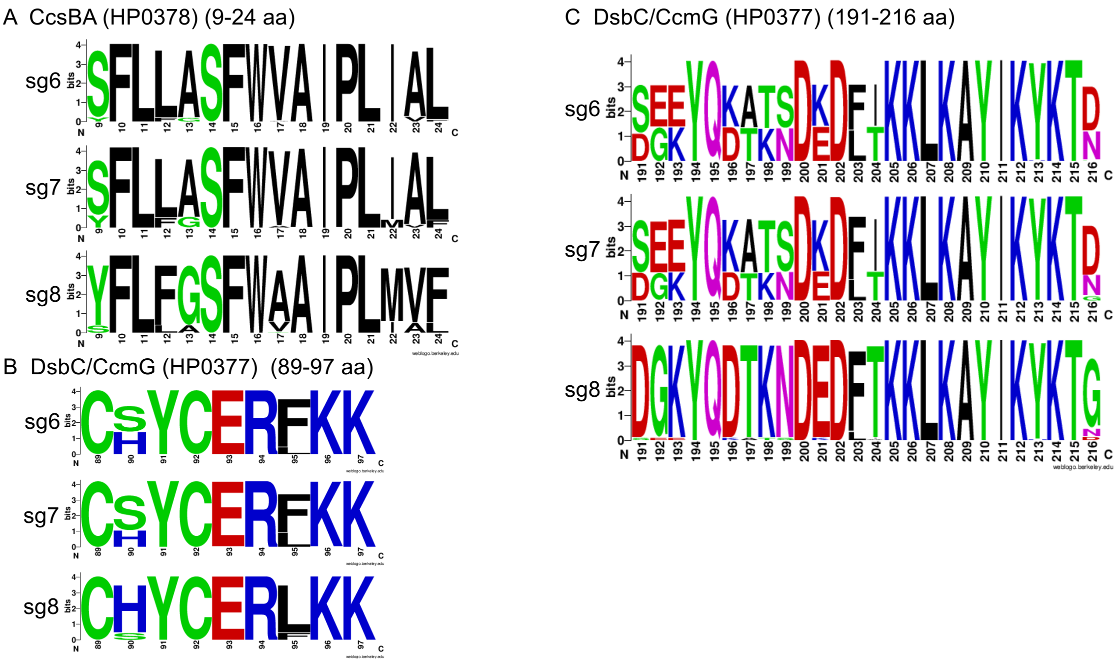

S5 Figure. WebLogo representation of non-synonymous mutations in CcsBA (HP0378) (A) and DsbC/CcmG (HP0377) (B, C).

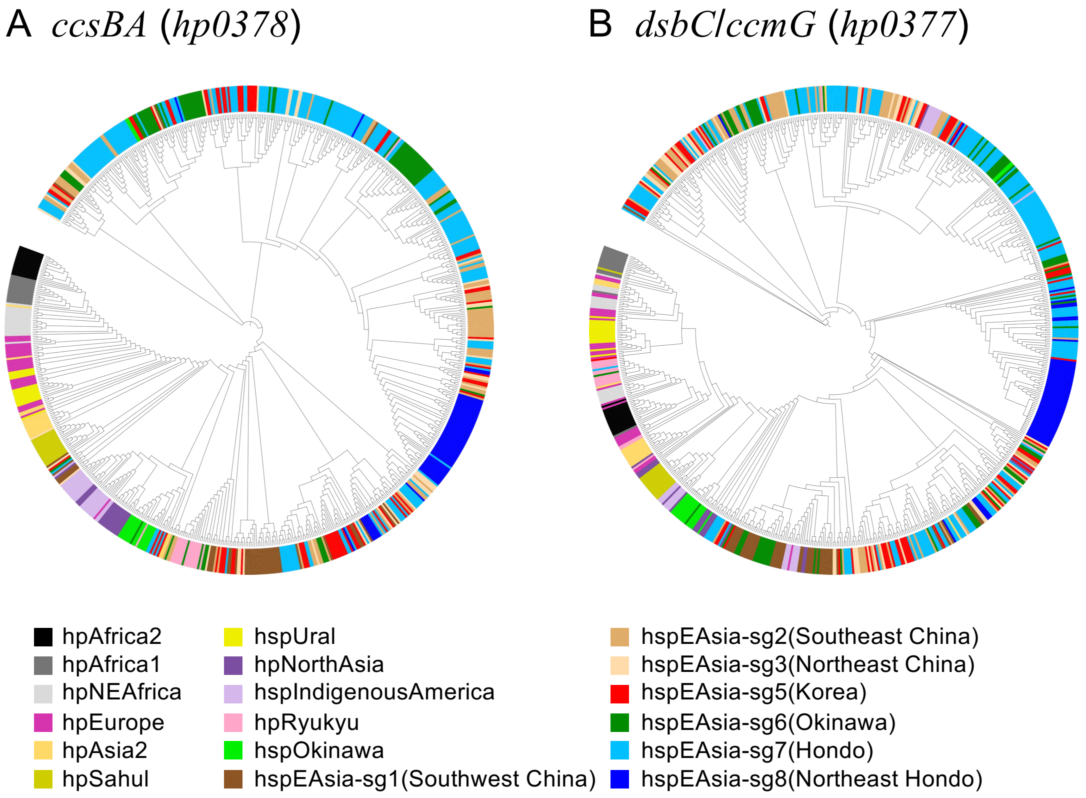

S6 Figure. The phylogenetic tree of differentiated genes in hspEAsia-sg8. (A) *ccsBA* (*hp0378*). (B) *dsbC/ccmG*(*hp0377*). Each strip is colored according to the populations defined by fineSTRUCTURE analysis.

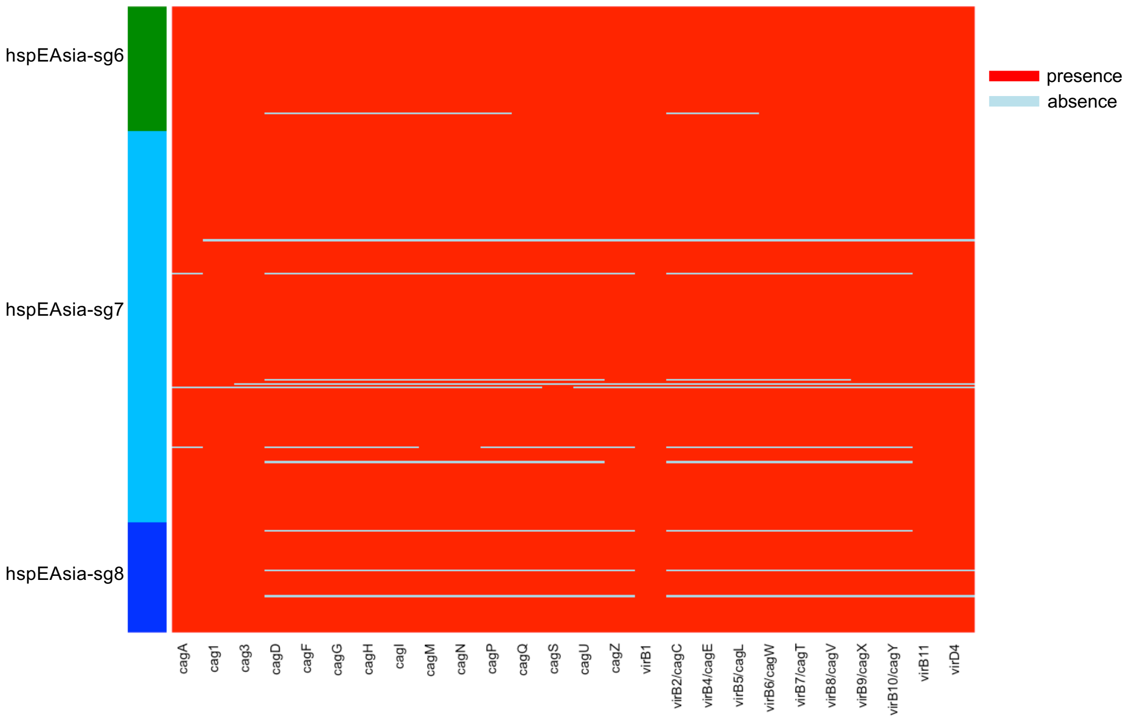

S7 Figure. The *cag*PAI genes presence/absence matrix among strains belonging to the Japanese hspEAsia subgroups. Each row shows gene presence (red) or absence (light blue) of each strain. The left bar represents the subgroups of each strain.
